# Reduction of intraoperative air leaks with Progel in pulmonary resection: a comprehensive review

**DOI:** 10.1186/1749-8090-8-90

**Published:** 2013-04-16

**Authors:** Clark Fuller

**Affiliations:** 1 Assistant Professor of Surgery, Surgical Director, Esophageal Center, Cedars-Sinai Medical Center, 8700 Beverly Blvd, Los Angeles, CA, 90048, USA

**Keywords:** Lung, Outcomes, Perioperative care, Surgery, Surgical equipment

## Abstract

Intraoperative alveolar air leaks (IOALs) occur in 75% of patients during pulmonary resection. Despite routine use of sutures and stapling devices, they remain a significant problem in the daily practice of thoracic surgery. Air leaks that persist beyond postoperative day 5 often result in increased costs and complications. Several large meta-analyses have determined that sealants as a class reduce postoperative air leak duration and time to chest drain removal, but these results did not necessarily correlate with a reduction in length of postoperative hospital stay. These analyses grouped surgical sealants together of necessity, but differences in efficacy may exist due to the differing product characteristics, study protocols, surgical procedures, and study endpoints. Progel, currently the only pleural surgical sealant FDA-approved for use in lung resection, has demonstrated efficacy and safety in two controlled clinical studies and superiority over standard air leak closure methods in reducing IOALs and length of hospital stay. This paper will review these findings and report on real-world experience with this recently approved pleural sealant.

## Introduction

Intraoperative alveolar air leaks (IOAL) occur in 75% of patients after pulmonary resection [1]. Despite the routine use of sutures and stapling devices, they remain a significant problem in the daily practice of thoracic surgery [2]. Air leaks that persist beyond the immediate postoperative period, defined as on or after postoperative day 5 [3], often result in increased medical and non-medical costs and complications, which may include longer drainage, greater postoperative pain, increased risk of infection, empyema, thromboemboli, and increased length of hospitalization [3-7].

The management of IOALs is best done intraoperatively. In addition to suturing the areas with visible leaks, several other intraoperative techniques have been developed to reduce postoperative IOALs. The most recent techniques are buttressing the staple line and using sealing agents to close leaks [3]. A Cochrane Database Review evaluating the use of surgical sealants in preventing or reducing postoperative air leaks after pulmonary resection found that surgical sealants were able to reduce postoperative air leaks [8]. Although this review, which included 16 randomized trials with 1642 patients, found that surgical sealants reduced postoperative air leaks and time to chest drain removal, it did not necessarily report leak reduction in association with a reduction in length of postoperative hospital stay. On that basis, systematic use of surgical sealants with the objective of reducing hospital stay was not recommended at the time of publication. Other authors, in reviewing the available data on lung sealants, have come to similar conclusions [3,9,10]. These reviews, which grouped all studied surgical sealants together of necessity, compared the use of a variety of surgical sealants with differing product characteristics, study protocols, surgical procedures, and study endpoints. This type of analysis does not permit the evaluation of safety and efficacy of any one agent and creates the impression that all sealants are the same and that an evaluation of the “class” is equal to an evaluation of each individual agent.

Progel® (Neomend, Inc., Irvine, CA), a polymeric biodegradable hydrogel sealant, is the only sealant FDA-approved for intraoperative use during pulmonary resection [11]. In the interest of determining whether or not Progel offers clinical benefits that outweigh risk and cost factors, particularly with respect to length of hospital stay, we review here the preclinical and clinical data for Progel. Additionally, we provide real-world insights regarding the use of this sealant and discuss novel applications and potential areas for future development.

## Review

A number of different materials have been used to develop surgical sealants [12]. These include fibrin glues, cyanoacrylate, GRF (gelatin-resorcinol cross-linked with formaldehyde), GRFG (GRF with glutaraldehyde), collagen, gelatin-based tissue adhesives, and polyurethane-based adhesives [12]. While each of these materials has some favorable sealant properties, they have been shown to have various disadvantages, including poor handling characteristics, need for special and cumbersome activation procedures, slow degradation, poor tissue adhesion, inflexibility, acute cytotoxicity, and chronic host-tissue responses induced by the degradation products [13,14].

**Figure 1:**
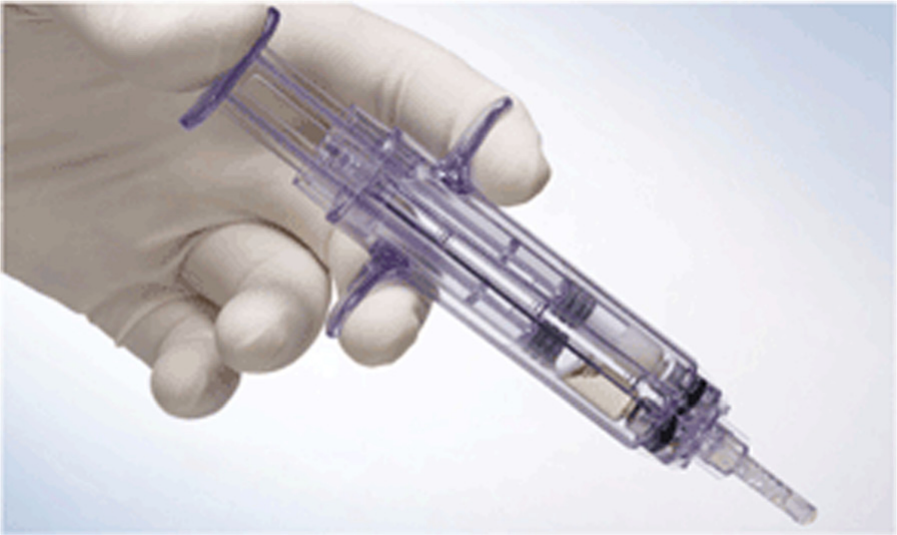
Progel surgical sealant applicator.

Progel does not require light for activation and was developed specifically for intraoperatively sealing alveolar air leaks resulting from surgical lung resection. It is produced by combining a polyethyleneglycol-based cross-linker, functionalized with succinate groups ([PEG-(SS)_2_]), with human serum albumin-USP just prior to usage. The cross-linker (PEG-SS_2_) and the albumin are sterilized and stored in separate cartridges within a single syringe applicator (Figure 1) to provide homogeneous mixing of the two liquid components and delivery onto the surface of the lung [12].

**Figure 2:**
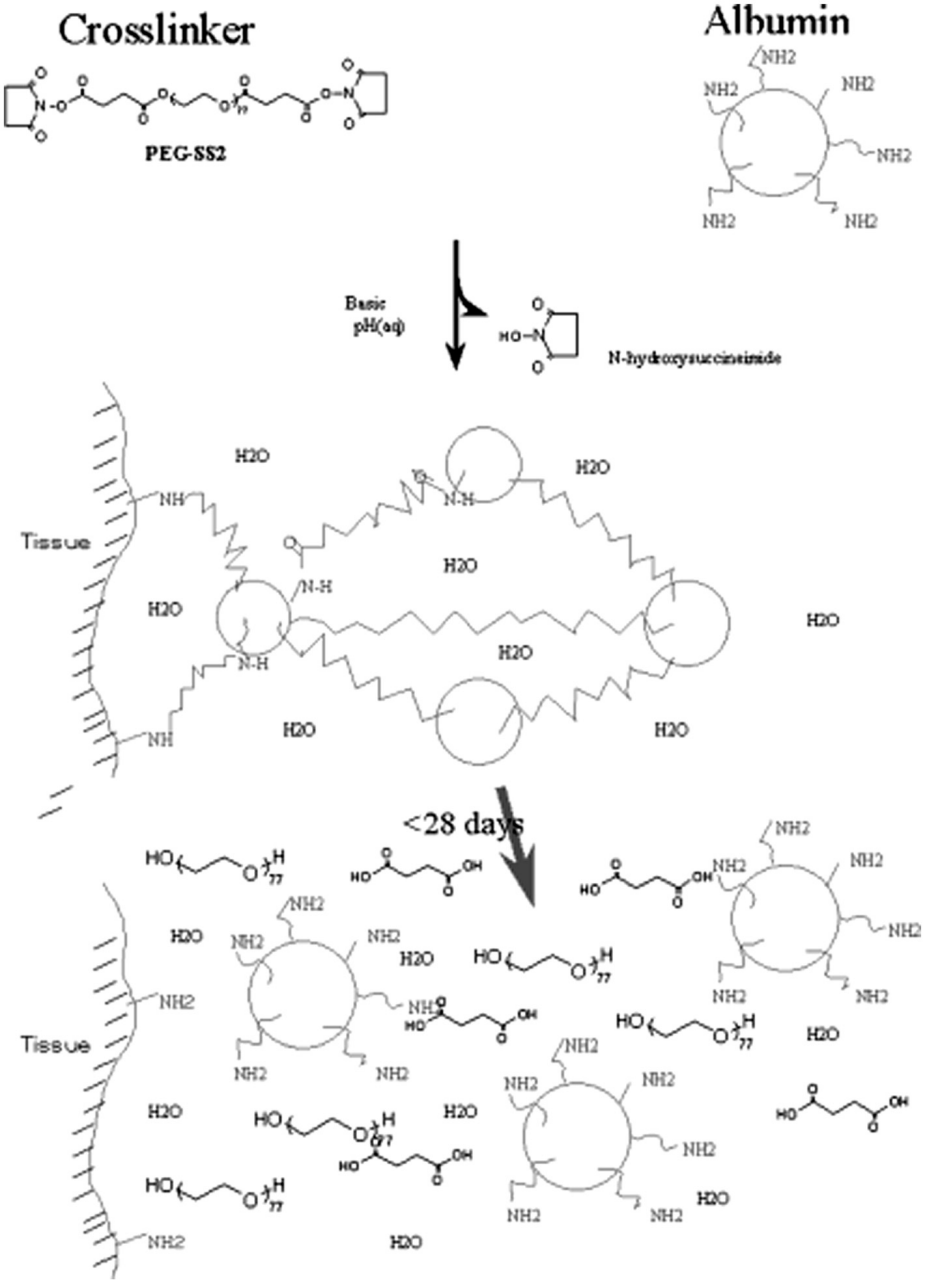
**Chemistry of starting materials and albumin-based hydrogel sealant (ABHS).** [Adapted with permission from Kobayashi *J Biomed Mater Res.* 2001]

In basic aqueous conditions, the lysine amine groups of the albumin react rapidly with the N-Hydroxysuccinimide (NHS) active esters at each end of the PEG-(SS)_2_ cross-link molecules. The reaction results in the formation of an amide bond between albumin and the cross-linker with the amine displacing N-Hydroxysuccinimide. The by-product of hydrogel formation is N-Hydroxysuccinimide, as shown in Figure 2. Succinate ester linkages remain in the formed hydrogel and serve as sites favorable for subsequent hydrolytic degradation of the gel [12]. Once mixed, the sealant polymerizes to form a cross-linked, clear, flexible hydrogel matrix that adheres to the lung tissue.

When the sealant contacts lung tissue, it conforms to the tissue by adhering to the microstructure of the lungs. The sealant stays in place and allows for the expansion and relaxation of the lung tissue until it biodegrades and is completely reabsorbed from the lung surface by 1 month after surgery [12-15].

### Preclinical studies

In a preclinical investigation, Kobayashi and colleagues compared the sealant properties of Progel and a fibrin glue (FG; Beriplast; Hoechest Marion Roussel Limited, Tokyo, Japan) in a rat lung incision surgical model [12]. Identical incisions (6 mm in length and 2 mm in depth) were made in the lobes of the lungs of rats in both the Progel and the FG groups. A 0.2–0.3-ml spray of each material was applied to the incision sites, according to the manufacturer instructions and the lungs were observed under 17–20 mmHg pressure while warm sterile saline was poured over the test site to determine the efficacy of each sealant in stopping air leaks. Burst pressures were measured at time 0 and at 3 days and 7 days postoperatively. The average burst pressures at time 0 for the FG and Progel groups were 30.8 ± 15.2 and 77.5 ± 19.1 mmHg, respectively. At Day 3, the average burst pressure of Progel (76.3 ± 15.8 mmHg) was still significantly higher (*p* < 0.05) than that of FG (60.0 ± 21.9 mmHg). At Day 7, no statistical difference was observed between the FG group (71.2 ± 18.6 mmHg) and the Progel group (88.8 ± 11.7 mmHg). In this study, Progel was found to have effective sealing properties after a single application in preventing air leakage in pulmonary surgery and was significantly superior to FG in the rat model [12]. In addition, histological examination of the lung tissue showed that in the Progel-treated animals, a homogeneous thin film was observed on the surface of the lung and that the adhesion was strong enough to withstand the lung inflation. At 3 days post-surgery, Progel seals were still intact and adhered well to the lung surface. No cell infiltration of the lung tissue was observed and the tissue response at the adhered surface was very mild and localized. At 7 days post-surgery, Progel could not be seen on the lung surface and infiltrates of various kinds of cells were observed at the site of original application. Macrophages and giant cells were observed at the site, and capillary invasion was also observed. At 14 days post-surgery at the incision site, there was no evidence of any adverse tissue reaction and a thin homogeneous film of Progel was observed on the surface of the lung with uniformly strong adhesion [12].

### Clinical studies

The clinical safety and efficacy of Progel in sealing intraoperative alveolar air leaks during pulmonary resection was evaluated in a multicenter, prospective randomized, controlled, clinical trial [16]. In this pivotal trial, the investigators demonstrated that Progel significantly reduced the number of patients who had a postoperative air leak and reduced the period of postoperative hospitalization following pulmonary resection.

Patients 18 years or older, who were scheduled for an open lung resection (lobectomy, bilobectomy, segmentectomy, wedge resection, or decortication), were evaluated for entry into the study. Patients had to have at least one significant intraoperative air leak (bubble size 2.0 mm in diameter) at the completion of the pulmonary resection for study inclusion.

The primary clinical efficacy endpoint was the proportion of patients who were air leak free following surgery through the 1-month follow-up period or duration of hospitalization, whichever was longer. The presence of air leaks during hospitalization was monitored by daily observation of the water seal chamber. Secondary efficacy endpoints included: the proportion of intra-operative air leaks in each group that were sealed or reduced; the proportion of patients that were free of air leaks immediately following surgery in the recovery room; the duration of postoperative air leak; chest tube duration; and the length of hospital stay. The incidence of adverse events related to the sealant that were reported during hospitalization and through the 1-month follow-up was the primary safety endpoint. In addition, changes in cellular and humoral immune response were monitored in both groups.

One hundred and forty-eight patients with significant intraoperative air leaks completed the trial. This included 95 patients treated with Progel after resections in which standard closure methods failed to result in air leak closure (sealant group) and 53 patients who received only standard air leak closure interventions (sutures and staples; control group).

**Figure 3:**
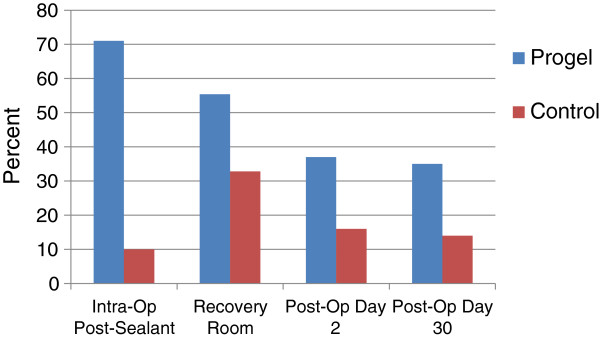
**Percent of patients air leak free versus time.** [Adapted with permission from Allen M, et al. 2004]

In the Progel group, 77% of air leaks were stopped after intraoperative application, while in the control group only 16% were stopped (*p* < 0.001). Throughout the study period, a higher percentage of patients were air leak free in the sealant group when compared to the control group (Figure 3). The proportion of patients who were air leak free in the recovery room was significantly higher in the sealant group (55.4%) compared with the control group (32.8%); (*p* = 0.002). Thirty-five percent (36/103) of patients remained air leak free following surgery through the 1-month follow-up in the sealant group compared with 14% (8/58) in the control group (*p* = 0.005). The benefits of Progel were seen irrespective of the number of intraoperative air leaks. If only one air leak was present, the success rate was 48% in the sealant group compared with only 20% in the control group. Additionally, if ≥ 3 leaks were observed, the success rate was 17% for the sealant group and 0% for the control group.

Progel, whether used adjunctively with sutures or staples or without, reduced or sealed an air leak 91% of the time compared with only 37% of the time in the control group (*p* < 0.001). A difference in the ability to seal air leaks was associated with the size of the air leak. Eighty-four percent of air leaks ≤5.0 mm were sealed with Progel compared with 17% in the control group. Fifty-eight percent of air leaks >5.0 mm were stopped by Progel compared with only 14% in the control group. The mean and median duration of postoperative air leaks was comparable in both groups, as was the median duration of chest tube drainage.

Importantly, the median length of hospital stay (LOS) was significantly shorter for patients in the sealant group. The median LOS for the Progel group was 6 days, while for the control group it was 7 days (*p* = 0.028).

The frequency of adverse events was similar in the sealant and control groups. Progel was not associated with any side effects and was non-immunogenic. The investigators reported that Progel is less complicated to use than other currently available sealants and it took a median of 6 minutes per patient to significantly reduce or eliminate air leaks.

The study design did not include patients without significant intraoperative air leaks and Progel was not used prophylactically and only applied to areas that were leaking air. Therefore, the rate of 35% for completely eliminating postoperative air leaks only includes patients who had significant (≥2.0 mm) intraoperative air leaks.

One possible flaw in the study design, and one which might explain the incongruity between the observed reduction in LOS and the non-significant difference in chest tube drainage, was that chest tube duration was measured in days rather than in hours. While speculative, it is possible that a difference in chest tube removal could have been found if the actual time of chest tube removal had been measured by a more sensitive hourly assessment.

In a single-center, retrospective chart review of prospectively collected data in 121 consecutive patients who underwent lung surgery with and without Progel, preoperative, operative, and 3-month postoperative data were evaluated [17]. The study included adult patients who underwent lung resection including lobectomy, segmentectomy, wedge resection, and decortications between May 2009 and August 2010. Patients treated without Progel were selected before May 2010, the time when Progel became available at the study site. Patients without intraoperative air leaks were excluded from the study.

When intraoperative air leaks were observed, standard surgical measures were applied, followed by Progel application. Progel was applied to sutures or staple lines in a discrete focal line initially and, after approximately one minute, reapplied in a mist to incorporate the surrounding 2-3 inches of tissue. In decortications, the method used was mist coverage over the entire decorticated surface.

All patients were followed for a minimum of 3 months following surgery and were assessed for the presence of postoperative air leaks, chest tube duration, and the length of hospital stay. Intraoperative and postoperative complications were reviewed and analyzed.

Seventy patients with lung procedures were included in the study (36 in the Progel group and 34 in the control group). Surgical procedures performed included 24 single wedge resections, 26 decortications, 9 lobectomies, 7 segmentectomies/bisegmentectomies and 4 other procedures. Segmentectomies and bisegmentectomies were more often performed in the Progel group. A chest tube was inserted in all patients in both groups to manage air leaks and drainage.

**Figure 4:**
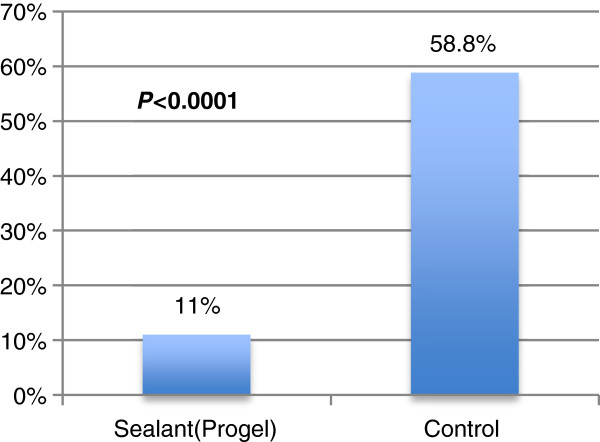
**Patients with postoperative air leak.** [Adapted from Klijian A. 2012]

Postoperative air leaks in the Progel group were significantly reduced. Only 11% (4/36) of the Progel group had postoperative air leaks compared to 58.8% (20/34) in the control group (*p* <0.0001) (Figure 4).

**Figure 5:**
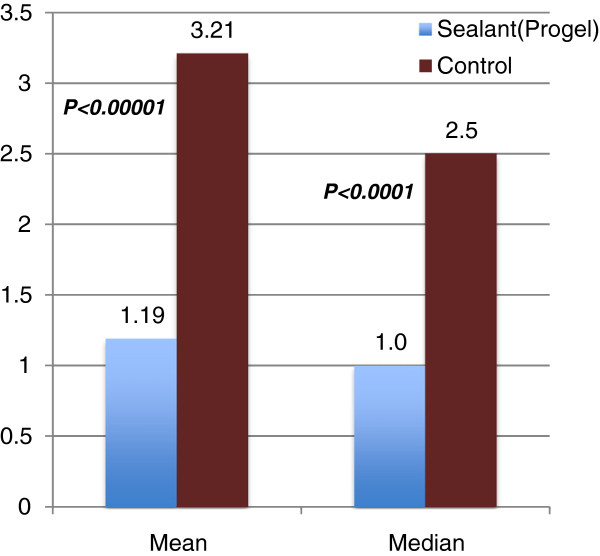
**Mean and median chest tube duration (days).** [Adapted with permission from Klijian A. 2012]

The duration of chest tube drainage was significantly reduced in the Progel group (Figure 5). The mean and median were 1.19 ± 0.52 and 1.0 days, respectively, in the Progel group versus 3.21 ± 2.14 and 2.5 days, respectively, in the control group (*p* < 0.0001), resulting in a difference of 2 days.

**Figure 6:**
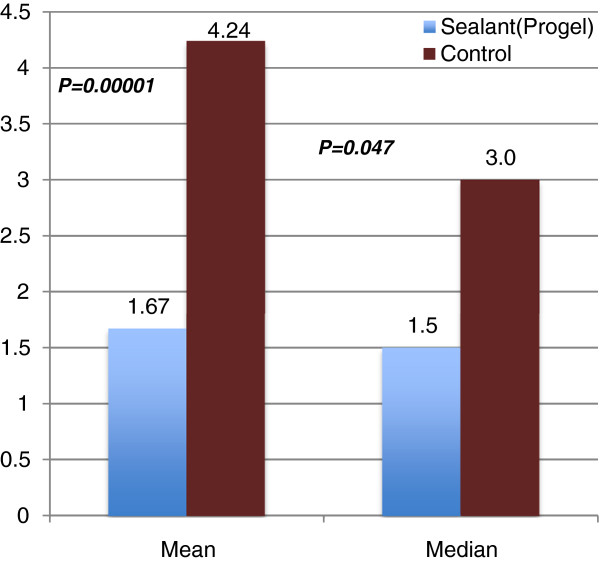
**Mean and median length of hospital stay (days).** [Adapted with permission from Klijian A. 2012]

In this study, as was observed in the pivotal clinical trial, LOS was significantly reduced in the Progel group compared with the control group (Figure 6). The mean and median were 1.67 ± 0.83 and 1.5 days, respectively, in the Progel group and 4.24 ± 2.13 and 3.0 days, respectively, in the control group (*p* = 0.047)

There was no statistically significant difference in complications between both groups. The results of this retrospective review show a significant reduction in intraoperative air leaks, postoperative air leaks, chest tube duration, and LOS with Progel used in addition to standard leak closure techniques in lung surgery when compared with standard management alone. In addition, these results confirm and extend those of Allen et al, further demonstrating a reduction in the duration of chest tube drainage.

In the most comprehensive review to date of the randomized clinical trials of surgical sealants in use for the intraoperative management of alveolar air leaks, Merritt and colleagues analyzed 16 studies [3]. A total of 1556 subjects undergoing lung resections were included in these studies. The surgical sealants studied included fibrin glues, an autologous fibrin sealant (Vivostat®), a human fibrinogen/thrombin coated collagen patch (TachoComb®), a photoactivated synthetic sealant (Advaseal®), bovine serum albumin/glutaraldehyde (Bioglue®), a polymeric hydrogel sealant (Coseal®), and Progel, a novel flexible, biodegradable polymeric hydrogel sealant. However, given the high degree of variability in study methodology, definitions of alveolar air leaks, and study endpoints, it was not possible to generalize about the efficacy of the sealants investigated. As a result, the reviewers, citing “inconclusive results” and increased costs, recommended against “indiscriminant” and nonselective use of sealants.

Other reviewers have come to a similar conclusion [1,8,9]. In the absence of standardized clinical trials and head-to-head studies, we are left with the need for surgeons to judge the value of each individual sealant separately on the basis of the data available to them. That judgment, from a practical clinical standpoint, should be based upon the incidence and duration of postoperative air leaks, the incidence of prolonged air leaks, the duration of chest tube drainage as a result of air leaks, possible adverse reactions, and the length of hospital stay. In addition, product- or device-specific characteristics such as ease of use, flexibility and adherence characteristics, burst strength, time required for application and sealing, toxicities and adverse events, and time to bioresorption should be considered.

From a systems perspective, the cost of use is often an important variable (i.e., cost per application, number of applications required, storage requirements, and burden of cost for “potentially preventable complications”). From the perspective of the patient and their caregivers, the effect on quality of life is a consideration (length of hospital stay, duration of chest tube drainage, risk of infection or other adverse events, possible effect on initiation of adjuvant chemotherapy) [18]. And, finally, the approval by the FDA for use in sealing air leaks during lung resections is an important indicator of safety and efficacy.

## Conclusions

In this evaluation of the published preclinical and clinical data on Progel, it has been demonstrated that Progel is an easy to apply, rapidly acting, highly adherent, flexible, tear-resistant, biodegradable, safe and effective surgical sealant. The results of the pivotal, randomized, controlled, multicenter clinical trial of Progel establishes its safety and efficacy in the management of intraoperative pulmonary air leaks [16]. Intraoperative air leaks were sealed in 77% of the Progel group compared with 16% in the control group (*p* < 0.001) and the Progel group had significantly fewer patients with postoperative air leaks compared with the control group (65% vs 86%, *p* = 0.005). The median LOS was 6 days for the Progel group compared with 7 days for the control group (*p* = 0.028). There were no observed differences in mortality, morbidity, duration of chest tube drainage, or immune responses between groups.

It is well recognized that the occurrence of prolonged air leaks increases the length of hospital stay [18]. In each of the two clinical studies of Progel, patients in the treatment group experienced a statistically significant reduction in length of hospital stay in comparison with the standard procedure control group [16,17]. In the multicenter, randomized study, Allen and colleagues found that Progel decreased the mean LOS by one day [16] and in the single-center retrospective study Klijian reported a 2.73 day reduction in mean LOS [17].

Varela and colleagues have reported that in a case series of 238 patients scheduled for pulmonary lobectomy (January 2001-December 2003) there were 23 patients (9.7%) with prolonged air leaks (PAL; ≥5 days) resulting in an increased length of stay [7]. The median LOS for all patients was 5 days. The median LOS for PAL cases was 10 days, compared with the non-PAL cases (*p* < 0.001). The estimated hospital stay for the 21 patients included in the analysis was 62.11 days. The cost of a one day hospital stay for lobectomy patients was €623.49. At the exchange rate of 1.4, which was current in 2005, the cost was $878.43 USD per day. When pharmacy costs were added in, the total cost per day for hospitalization was $890.51. At a median of 5 days increased LOS, we calculate the excess cost per PAL patient was $4452.55.

While such a study has not been reported for patients treated with Progel, we may assume that with a demonstrated median reduction in LOS of 1-2.73 days and a similar cost per day of postoperative hospitalization, the intraoperative use of Progel could result in a savings of $890 to $1610 in 2012 dollars. This represents a considerable monetary saving, and may also result in conservation of surgeon time, less in-hospital follow-up, potential reduction in patient risk of exposure to hospital-acquired infection, and a probable increase in the quality of life for patients and their caregivers.

Reviews of lung surgical sealants have indicated that their greatest value may reside in their intraoperative use in patients at highest risk of prolonged air leaks (i.e., patients with COPD, severe emphysema, those with FEV_1_ less than 35% predicted, and those with large air leaks) [3,19].

In conclusion, Progel is currently the only FDA approved surgical sealant for use in lung resection. The safety and efficacy of Progel has been demonstrated in two controlled clinical studies. It has been shown to be superior to standard intaroperative air leak closure methods in reducing intraoperative air leaks, postoperative air leaks, and length of hospital stay. We believe that the data reviewed here argue in favor of the use of Progel in lung resection surgery, in which there are air leaks that are difficult to close using standard suture and staple methods. We also suggest that a study of Progel effectiveness in the reduction of prolonged air leaks in high-risk patients seems to be warranted at this point.

## Competing interests

Dr. Fuller is a consultant to Neomend, Inc., maker of Progel Pleural Air Leak Sealant.
